# Are clinician-administered ataxia scales truly culture-free? Rethinking contextual validity in diverse populations

**DOI:** 10.3389/fneur.2026.1991845

**Published:** 2026-10-23

**Authors:** Hina Narzari, Ravi Yadav, Achal Kumar Srivastava, Pramod Kumar Pal, Divyani Garg

**Affiliations:** 1 Department of Neurology, All India Institute of Medical Sciences, New Delhi, India; 2 Department of Neurology, National Institute of Mental Health and Neurosciences (NIMHANS), Bangalore, India; 3 Department of Neurology, St. John’s Medical College, Bangalore, India

**Keywords:** contextual adaptation, cross-cultural adaptation, hereditary cerebellar ataxia, ICARS, measurement equivalence, SARA

## Abstract

Hereditary cerebellar ataxias are genetically and phenotypically heterogeneous progressive neurodegenerative disorders characterized by substantial functional impairment. With the emergence of disease-modifying therapies and gene-targeted clinical trials, standardized and reliable clinical outcome measures are essential for assessing disease severity and progression. Among the available scales, two are widely used by clinicians to assess ataxia: The Scale for the Assessment and Rating of Ataxia (SARA) and the International Cooperative Ataxia Rating Scale (ICARS). Given considerable linguistic, educational and sociocultural heterogeneity across the globe, we opine that it may pose challenges to the uniform application and cross-cultural comparability of these instruments. Published translations and adaptations were also examined to determine whether cultural adaptation extended beyond linguistic translation. Among all the identified adaptations, reported processes predominantly comprised forward and backward translation, expert review and assessment of linguistic, conceptual or cultural equivalence. These findings suggest that linguistic translation alone may not be sufficient to ensure that clinician-rated ataxia scales retain equivalent meaning and measurement across diverse populations. We therefore introduce the concept of “contextual validity” emphasizing that language, educational heterogeneity, literacy, graphomotor familiarity, cultural practices and testing environments that may influence performance independently of cerebellar dysfunction. This perspective outlines considerations for developing contextually adapted and psychometrically validated Indian-language versions of SARA and ICARS to improve measurement accuracy and support equitable clinical assessment and participation in global clinical trials.

## Introduction

Ataxia is a clinical syndrome arising from dysfunction of the cerebellum and/or its afferent and efferent pathways (1). It is characterized by deficits in the coordinated execution of gait, limb movements, oculomotor function, speech and stance. It occurs either in isolation or in combination (1–4). Among the myriad etiologies of ataxia, hereditary cerebellar ataxias constitute a genetically and phenotypically heterogeneous spectrum of progressive neurodegenerative disorders that confer substantial functional impairment. While hereditary ataxias are individually rare, they collectively affect an estimated one in 10,000 people worldwide and represent an important cause of neurological disability across diverse populations (3). India is projected to have one of the world’s largest populations (5) and recent advances in molecular genetics have transformed the diagnostic landscape of hereditary ataxias (2, 6, 7). The emergence of disease-modifying therapies and gene-targeted clinical trials, alongside large observational cohorts (8, 9) have heightened the need for reliable and standardized clinical outcome measures capable of accurately monitoring the disease severity and its progression (8, 10, 11).

Several clinician-rated instruments have already been developed to quantify the severity of ataxia, including the International Cooperative Ataxia Rating Scale (ICARS) (10), the Scale for the Assessment and Rating of Ataxia (SARA) (11), the Brief Ataxia Rating Scale (BARS) (12) and disease-specific instruments such as the Friedreich Ataxia Rating Scale (FARS) (13). Among these, SARA and ICARS remain the most extensively validated and widely adopted in studies or trials (14, 15). ICARS was developed in 1997 by the Ataxia Neuropharmacology Committee of the World Federation of Neurology with its principal development center in Lyon, France and SARA was developed in 2006 at the University Hospital Bonn, Germany. Despite their widespread adoption in clinical practice, both scales were developed and have been predominantly evaluated in populations that differ substantially from the sociocultural and linguistic diversity encountered in India. Many cross-culturally adapted/translated versions of SARA and ICARS also exists. Among the adaptations which could be identified, most were developed in settings with a predominant national language or a structured bilingual or multilingual environment with few countries demonstrating regional or minority-language diversity. It could be noted that all the reported processes usually comprised of forward and backward translation followed by expert review for linguistic, conceptual or cultural equivalence. Among the adaptations identified in our review, we found limited evidence of substantive modification of assessment tasks or procedures specifically to address contextual factors beyond linguistic, conceptual or cultural equivalence (Table 1). India on the other side, presents a particularly complex adaptation context because multiple major regional languages coexist with substantial variation in literacy, educational attainment and cultural practices. Therefore, the applicability of these instruments within the Indian context warrants careful consideration. The availability of culturally adapted and psychometrically validated Indian-language versions of SARA and ICARS would facilitate consistent assessment across centers, improve data quality and strengthen India’s participation in international collaborative research. Assessment of patients in India is often riddled with challenges of complex assessment environment characterized by remarkable linguistic, educational and cultural diversities.

**Table 1:** International translations and adaptions of SARA and ICARS.

Region	Country	Scale	Year of adaptation	Translated language and linguistic setting	Literacy/Educational attainment	Translation or adaptations made
Europe	Russia	SARA	2026	Russian; predominant (24, 25); approximately 300 languages are used in the Russian Federation (24)	Adult literacy rate was 99.93% among 15+ years population in 2021; Educational attainment rate who completed primary education or higher of people aged 25 + was 100% in 2024 (26)	Forward and backward translation, Expert review and linguistic/cultural adaptation. Adaptation primarily concerned translation and linguistic/cultural equivalence (27)
Europe	Italy	SARA	2025	Italian; predominant, dialects and minority languages are also spoken (28)	Adult literacy rate was 99.35% among 15+ years population in 2019; Educational attainment rate who completed primary education or higher of people aged 25+ was 100% in 2023 (26)	Forward and backward translation, expert review for conceptual equivalence, expert assessment of content. Wordings refined for task clarity. Suggested age-adjusted cutoff values are important (29)
South America	Argentina	SARA	2025	Spanish; predominant (30); indigenous languages (31)	Adult literacy rate was 99.14% among 15+ years population in 2020; Educational attainment rate who completed primary education or higher of people aged 25+ was 88.44% in 2022 (26)	Forward and backward translation, expert review for conceptual equivalence, expert assessment of content (32)
Asia	Thailand	SARA	2024	Thai; national language which is also standardized (33); more than 70 indigenous languages (34)	Adult literacy rate was 91.10% among 15+ years population in 2022; Educational attainment rate who completed primary education or higher of people aged 25+ was 75.45% in 2024 (26)	Two independent forward translations, reconciliation, two back-translations and expert review for semantic, idiomatic, experimental and conceptual equivalence (4)
Asia	Iran	SARA	2024	Persian; official (35), predominant (36); numerous other languages (36)	Adult literacy rate was 88.92% among 15+ years population in 2023; Educational attainment rate who completed primary education or higher of people aged 25+ was 86.88% in 2023. (26)	Forward and backward translation with linguistic and cultural equivalence assessment, expert review for face and content validity (37)
Europe	German-speaking region (Germany, Austria, Switzerland)	SARA	2024	German; official in Germany (38) with recognized regional and minority languages also spoken and protected (39); in Austria with minority languages also having official status in certain regions of Austria (40) and one of Switzerland’s four official languages and the most widely spoken (41)	Among people aged 25+, educational attainment rate for those who completed primary education or higher was 100% in Germany and Austria in 2023 and 99.62% in Switzerland in 2024 (26)	Multiple forward and backward translations, expert committee review and comprehensibility assessment by clinicians from Germany, Austria and Switzerland. Wording and task terminology were refined; gender-neutral wording (“Person”) was adopted (42)
Asia	Korea	ICARS	2023	Korean; official, predominant (43) with regional dialects (44)	Adult literacy rate was 97.97% among 15+ years population in 2008; Educational attainment rate who completed primary education or higher of people aged 25+ was 97.88% in 2024 (26)	Forward and backward translation, reviewed, errors corrected with consideration of Korean cultural differences and the unspecified size of the Archimedes spiral were identified but not corrected (45)
North America	Canada	SARA	2023	French; English and French are Canada’s two official languages (46)	Educational attainment rate who completed primary education or higher of people aged 25+ was 96.43% in 2024 (26)	Forward and backward translation and cross-cultural validation, translation reviewed for linguistic equivalence and comprehensibility by experts (47)
Europe	Czech Republic	SARA	2022	Czech; predominant, it is the mother tongue of 92.1% of the population who are listed with one mother tongue; minority/immigrant languages exist (48)	Educational attainment rate who completed primary education or higher of people aged 25+ was 99.89% in 2023 (26)	Forward and backward translation with two translators, supervisor consultation, and usability assessment by therapists and 9 patients (49)
South America	Brazil	ICARS	2018	Portuguese; official, predominant (50); indigenous and immigrant/minority languages exists (51). Among these indigenous population 68.54% were reported could speak Portuguese (52)	Adult literacy rate was 94.74% among 15+ years population in 2024; Educational attainment rate who completed primary education or higher of people aged 25+ was 79.57% in 2024 (26)	Forward and backward translation, expert committee review and pilot testing; final version considered culturally appropriate for Brazilian patients (53)
Asia	Türkiye	SARA	2017	Turkish; official (54) and predominant since it is the mother tongue of 90% of the population (55); around 70 other minority languages and dialects also exists (55)	Adult literacy rate was 97.26% among 15+ years population in 2021; Educational attainment rate who completed primary education or higher of people aged 25+ was 86.23% in 2023 (26)	Forward and backward translation and expert review. Discrepancies were corrected to establish linguistic and cultural equivalence (56)
Asia	Türkiye	ICARS	2017	Turkish		Forward and backward translation and expert review; discrepancies between original and Turkish SARA were corrected to establish linguistic and cultural equivalence. (56)
Asia	Korea	SARA	2014	Korean		Forward and backward translation, expert review for Korean linguistic and cultural equivalence. (57)
Asia	China	SARA	2013	Chinese (Putonghua/Standard Mandarin); Common language used nationwide; regional and minority languages are also spoken (58). Standard Chinese is used by 80.72% of the population (59)	Illiteracy 2.67% in 2022 (59); Adult literacy rate was 96.74% among 15+ years population in 2020; Educational attainment rate who completed primary education or higher of people aged 25+ was 95.84% in 2020 (26)	Forward and backward translation with linguistic/cultural equivalence considered. Adapted for use within the Chinese clinical setting (60)
South America	Brazil	SARA	2010	Portuguese		Forward and backward translation, expert review and pilot testing to establish linguistic/cultural appropriateness (61).
Asia	Japan	SARA	2009	Japanese; it is predominantly spoken, with non-native speakers and Japanese-language learners are also present in Japan (62)	Educational attainment rate who completed primary education or higher of people aged 25+ was 99.59% in 2020 (26)	Forward and backward translation with linguistic/cultural equivalence considered (63)

While translation of clinician-administered outcome measures into Indian languages represents an essential first step, translation alone may not ensure that an instrument measures the same underlying construct across diverse populations. The concept of contextual validity is broader and encompasses accurate translation along with the ability of the scale to retain conceptual, functional and psychometric equivalence across diversity of language, education, cultural practices and lived experiences. Performance on clinician-administered ataxia scales may be influenced by contextual factors that are unrelated to cerebellar dysfunction, introducing systematic and/or random measurement errors and potentially compromising comparability across individuals, centers, countries and populations. We propose that contextual validity of a clinician-administered performance measure should be considered at multiple levels: linguistic equivalence of instructions, conceptual equivalence of the construct being assessed, functional equivalence in the ability to perform the task, psychometric equivalence of item and scale performance, and implementation equivalence across examiners and testing environments. A scale may therefore be linguistically well translated yet remain contextually non-equivalent if task familiarity, examiner interaction or testing conditions systematically influence performance. We demonstrate these points, using the Indian context as exemplary.

## Clinician-rated performance scales and cultural context

Clinician-administered outcome measures that are based on performance of specific tasks (such as SARA and ICARS) are perceived to be objective assessments as they are reliant on direct observation of performance of tasks rather than on self-report by patients. As a result, these outcome measures are assumed to be less affected by cultural factors than patient-reported outcome measures (PROM).

However, the administration of clinician-rated scales actually represents a complex interaction between the examiner, the participant and the testing environment. Successful completion of many performance-based tasks depends not only on neurological function but also on comprehension of verbal instructions provided by the examiner, overall familiarity of the assessed person with the requested activity, examiner demonstration, participant motivation and cultural patterns of communication and behavior. In SARA and ICARS, several tasks that assess gait, stance, finger chase, heel-shin, rapid alternating hand movements (pronation-supination) and speech, require standardized verbal instructions and active participant cooperation. Instructions may be translated, paraphrased, explained or supplemented with physical and/or video demonstrations to improve understanding, particularly in diverse lingual settings. Although such adaptations are frequent and sometimes unavoidable in clinical practice, they may alter task interpretation or execution and introduce variability that is unrelated to cerebellar dysfunction. The participant’s exposure to specific motor tasks, educational experiences and sociocultural norms may influence their performance parallelly.

Recognizing that clinician-rated performance scales may be context-dependent has important implications for clinical practice and research. Cross-cultural adaptations should aim to translate assessment instructions while preserving equivalence across diverse populations. This broader perspective forms the basis of contextual validity (Figure 1). In this context, an outcome measure is expected to perform equivalently and consistently despite differences in language, education, culture and lived experience.

**Figure 1:**
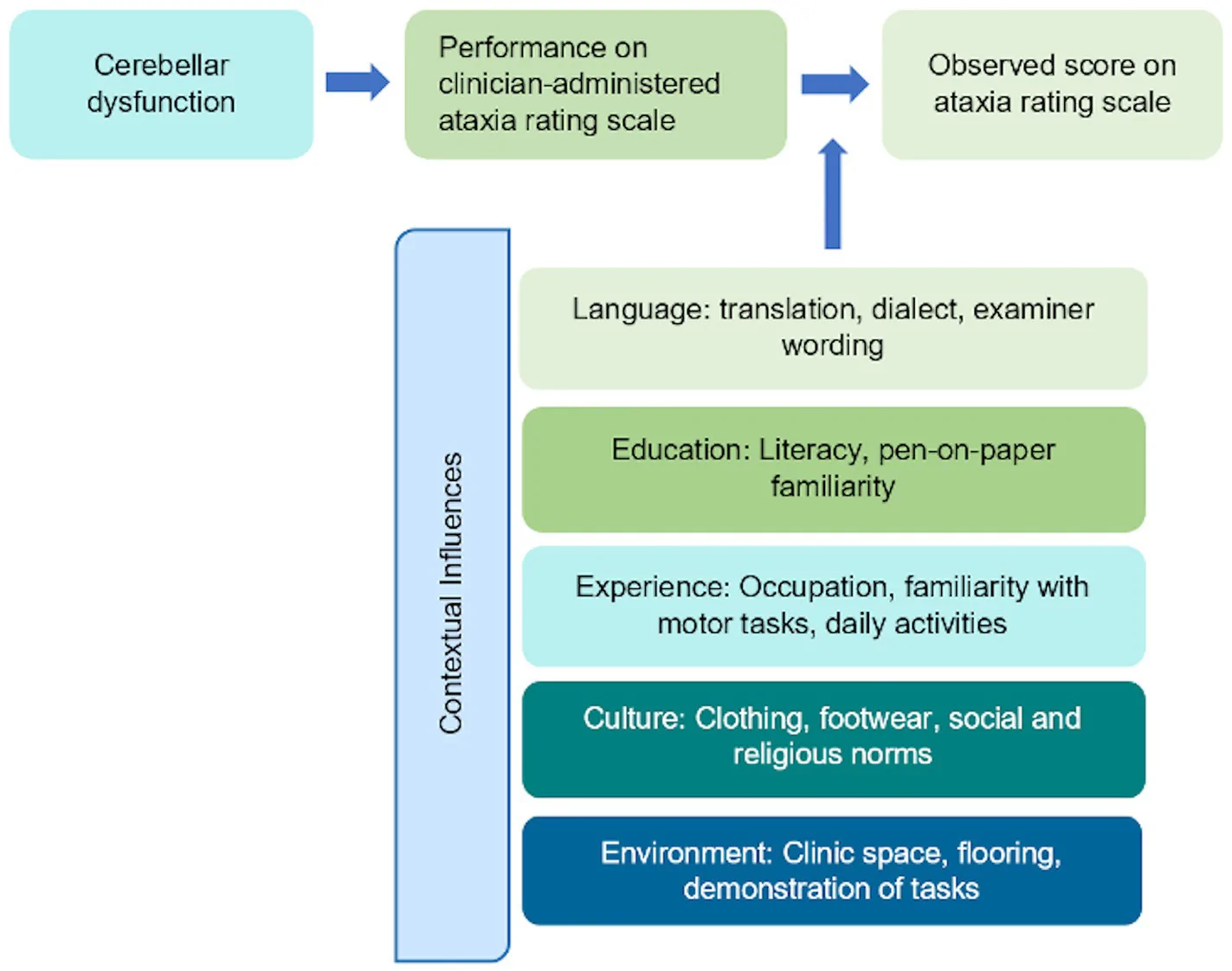
Contextual determinants of clinician-administered ataxia assessment.

## Linguistic diversity

Patients and examiners may not always share a common primary language, particularly in tertiary referral centers serving geographically diverse populations. For instance, in India, there are 19,569 reported mother tongues that were rationalized into 1,369 linguistic varieties, 121 major languages, and 22 constitutionally recognized scheduled languages (16). Speech assessment also presents an additional challenge beyond language. SARA and ICARS include assessment of speech characteristics such as fluency, clarity of articulation and phonation, yet speech production varies substantially across regions, with differences in accent, pronunciation, prosody and articulation occurring even among speakers of the same language and sometimes across relatively small geographic areas. These features may represent habitual patterns which are acquired through lifelong exposure to a particular language, dialect and speech community rather than neurological impairment. When an examiner is unfamiliar with the patient’s regional speech pattern he/she may potentially misinterpret unfamiliar pronunciation, accent or articulation as abnormal speech, especially while assessing features such as dysarthria, fluency or phonation. This highlights the need for culturally and linguistically informed guidance when assessing speech-related items in SARA/ICARS so that it becomes easier to distinguish genuine neurological abnormalities from normal regional or dialectal variation. Even though SARA and ICARS are performance based clinical assessment scales, several items including stance, finger-chase, fast alternating hand movements, heel-shin slide and speech tasks depend on consistent verbal instructions and participant cooperation, making conceptual equivalence across languages an essential component of reliable outcome assessment (17). In the absence of culturally adapted and validated Indian-language versions of both scales, administration may rely on examiner-mediated, real-time translation of the original English instructions into a mutually understood language. This may result in variations in phrasing, terminology and explanation across assessors and study sites potentially affecting participant comprehension, reducing inter-rater reliability and compromising the reproducibility and comparability of outcome measurements (17).

## Educational heterogeneity

Educational heterogeneity may further complicate these challenges. According to the Periodic Labor Force Survey (PLFS) 2023-24, approximately one-fifth of India’s population aged ≥7 years is not literate (19.1%), with the proportion nearly twice as high among females (25.2%) as males (12.8%). Furthermore, the PLFS 2025 reports that 32.2% of individuals aged ≥15 years have not attained at least secondary education with marked rural–urban (38.1% vs. 20.3%) and sex-based (39.7% in women vs. 24.5% in men) gradients. Similarly, the National Family Health Survey (NFHS-6, 2023-24) reports that 53.6% of women and 45.4% of men have not completed 10 or more years of schooling nationally, with pronounced rural–urban differences (60.3% in rural women vs. 38.5% in urban women; 50.1% in rural men vs. 36.0% in urban men).

These educational differences may also extend beyond comprehension of verbal instructions to familiarity with task demands. Several items in SARA and ICARS require participants to understand and execute instructions consistently. These tasks may not be a part of routine daily activities. For instance, the spiral drawing task in ICARS requires participants to reproduce a template spiral using a pen, a task that assumes a degree of familiarity with pen-and-paper or graphomotor activities acquired through routine writing or drawing. Individuals with limited or no formal education, those engaged primarily in manual occupations, and from rural and socio-economically disadvantaged settings may have had limited opportunity to structured drawing and writing tasks and other pen-and-paper activities. Additionally, increasing reliance on smartphones and touchscreen devices may have reduced exposure to graphomotor tasks in a subset. Therefore, performance on graphomotor tasks could partly reflect graphomotor familiarity, representing a potential source of measurement variability that warrants empirical evaluation. During cross-cultural adaptation, standardized task demonstrations or modified formats may enable individual tasks to maintain equivalence across educational level and social backgrounds.

## Cultural diversity

Beyond language and education, cultural practices and daily living habits may also influence the administration and interpretation of clinician-rated ataxia scales. India’s cultural diversity is mirrored in 4,635 documented communities with distinct customary practices, traditional attire, occupational lifestyles and sociocultural practices (18). Garments such as sarees, mekhela chadors, dhotis, lungis, mundus, ghagras, burqas and other region-specific draped full-length clothing remain widely worn in many parts of the country (19). During gait and stance assessment as well as tests such as the heel–knee test, such garments may influence stride length, lower-limb excursion or balance performance independent of cerebellar dysfunction. As neither SARA nor ICARS provides explicit recommendations regarding attire during assessment, variability related to clothing may introduce avoidable inconsistencies in performance across participants and study sites. In some cultural or religious settings, participants may prefer not to modify traditional attire during clinical assessment because of personal, cultural or religious considerations. Hence, the extent to which clothing influences performance may vary unless harmonized assessment procedures are established.

Similarly, participants may be assessed either barefoot or while wearing a wide variety of footwear, including slippers, sandals or shoes, depending on regional customs, personal preference and socioeconomic circumstances. As footwear can influence gait characteristics and postural stability, the absence of clear recommendations regarding the type of footwear in SARA and ICARS may introduce additional variability unrelated to neurological impairment.

## Local logistics

Additional considerations include surface variability and space constraints. For tandem gait, a patient unfamiliar to the polished or tiled hospital floor or carpeting may face difficulties. Many hospitals are unable to provide a 10 m walking space, and walking examination may be performed in crowded outpatient areas, influencing gait acceleration and turning.

The assessment of oculomotor function, which is a key item on the ICARS scale, relies on examiner techniques and interpretation. The evaluation of gaze-evoked nystagmus, pursuits, saccades require standardized target positioning, target velocity, distance from target and illumination, which are not specified explicitly. Hence, variability in scoring oculomotor function may arise. These aspects are important in multi-centric trials where differences in rating scores may contribute to between-site differences. In resource-constrained settings, standardized examiner training and pragmatic approaches to oculomotor assessment may be particularly important.

## Occupation-related factors

Another crucial contextual factor is the patient’s occupation. Requirements for coordination may vary for skilled activities such as driving, cooking, tailoring or hairstyling where manual coordination is crucial. For a homemaker, trunk and lower limb coordination may be equally relevant. Even among these activities, further finesse may be essential beyond that tested by finger-nose testing or pronation-supination tasks, for actions such as threading a needle, fine-chopping vegetables, using scissors etc. It may be useful to evaluate how scores on coordination tests translate in real-life in the performance of different activities such as eating with a spoon, placing hands inside a sleeve, shaving, cutting hair (for a barber) or cloth (for a tailor). Rather than replacing standardized coordination tasks with occupation-specific assessments, future studies could examine how performance on standardized ataxia scales translates into ecologically relevant activities across occupational and social contexts. This may provide complementary information regarding functional impact without compromising the comparability of the core neurological outcome measure.

Table 2 provides pragmatic examples from clinical practice demonstrating the influence of context-related factors on performance on ataxia rating scales.

**Table 2:** Clinical examples demonstrating the potential influence of contextual factors on rating scores.

Scale domain	Contextual factor/s	Potential influence on score	Possible mitigation
Real-time translation of instruction	Language	Different interpretations	Standardized multilingual scripts
Demonstration of tasks	Examiner’s instructions	Inter-rater variation	Training and certification for harmonization
Spiral drawing/pen-and-paper tasks	Education, socioeconomic status, occupation	Graphomotor familiarity may influence performance on score	Pilot testing, cognitive interviews
Finger-to-nose, finger chase and pronation-supination movement testing	Familiarity with task	Previous exposure to structured tasks may improve performance on scores	Standardized demonstrations
Gait (including tandem gait assessment) and heel–knee-shin/knee-tibia test	Clothing (Saree, dhoti, lungi etc.)	Gait restriction or modification due to apparel	Standardized guidance on attire or adaptation to minimize influence of attire
Gait (including tandem gait assessment)	Barefoot versus shoes	Balance differences	Uniform footwear protocol
Gait (including tandem gait assessment)	Clinic corridor length	Gait variability	Standardized testing area/space
Eye movement testing	Examiner technique, illumination, target specifications, interpretation	Inter-rater variation	Standardized protocol for testing
			Item removal may be considered in abbreviated or adapted scales

## Implications for multinational clinical trials

Clinician-administered ataxia rating scales such as SARA are being increasingly employed as primary or secondary outcome measures in natural history studies and interventional trials evaluating emerging disease-modifying therapies for hereditary ataxias. Such studies assume that a given score represents an equivalent degree of neurological impairment irrespective of the participant’s linguistic, cultural or educational background. However, if contextual factors systematically influence the performance of specific tasks, the observed differences in outcome scores may be reflective of measurement artifacts rather than true biological variation. For instance, a variability by the listener in the interpretation of verbally administered instructions or in writing or drawing based tasks, may lead to random measurement errors across centers. These may, in turn, influence estimates of disease progression, effects of treatment, sample size calculation and even the interpretation of clinically meaningful change in scores. Across-site differences due to these factors may complicate geographical comparisons.

Importantly, these considerations remain relevant even when SARA or ICARS has already been pre-specified as an outcome measure. Examination of item-level performance, between-center variability and patterns of inconsistent scoring may help identify whether some observed variation could reflect differences in administration or testing context rather than biological differences. This does not imply retrospective modification of established outcome measures, but rather recognition of contextual sources of measurement variability when analyzing and interpreting multi-center trial data. For ongoing multi-center studies, several sources of contextual variability may be addressable without changing the outcome measure. Standardized multilingual instructions, examiner training and certification, harmonized testing procedures could improve consistency across sites. Such pragmatic measures may be particularly useful when recruitment spans populations with substantial linguistic, educational or cultural heterogeneity. Thus, the immediate application of contextual validity principles need not require replacement of established scales, but may instead involve improving the consistency of their administration and interpretation.

In India, these considerations are particularly pertinent, which is expected to play an increasingly relevant role in global ataxia research as a result of a large affected population, expanding molecular diagnostic capacity and capability and increasing participation in international studies. Ensuring that clinician-administered outcome measures are contextually adapted and psychometrically equivalent across Indian languages and cultural settings will therefore not only improve national research quality but also strengthen the reliability and interpretability of data contributed to multinational registries and clinical trials. Incorporating principles of cross-cultural adaptation, measurement invariance testing and differential item functioning into trial methodology may help ensure that observed differences in clinical outcomes reflect disease biology and treatment response rather than contextual influences on scale performance.

## Future directions

Beyond linguistic translation, future work must move toward the development of contextually applicable and valid clinician-administered outcome measures while preserving psychometric validity across diverse populations and contexts (Figure 2). This is achievable through multi-centric collaborations that involve neurologists, rehabilitation specialists, psychologists, linguists, and patient representatives. The aim should be to develop standardized protocols for administration of culturally appropriate adaptations while simultaneously remaining true to original constructs of the scales. Validation studies should expand beyond conventional psychometric validation to assess scale responsiveness across diverse linguistic and cultural settings. It is also crucial to develop harmonized multilingual instructions, certification programs and training modules to further enhance consistency across centers and reduce inter-rater reliability. AI-enabled multilingual platforms incorporating standardized versions of SARA and ICARS could facilitate consistent administration across different languages by providing accessible, language-specific instructions while retaining clinician-led scoring. Such tools could reduce language barriers, ease administration and promote wider adoption of these scales among neurologists and movement-disorder specialists. Speech assessment may require particular attention because pronunciation, accent, prosody and dialectal characteristics may overlap with features that clinicians interpret as dysarthria. AI-assisted speech analysis could potentially provide quantitative and reproducible measures of acoustic and temporal features and may assist in distinguishing neurological speech abnormalities from linguistic or dialect variation. However, such approaches would require rigorous validation across languages, dialects, educational backgrounds and recording environments before they could be considered robust outcome measures.

**Figure 2:**
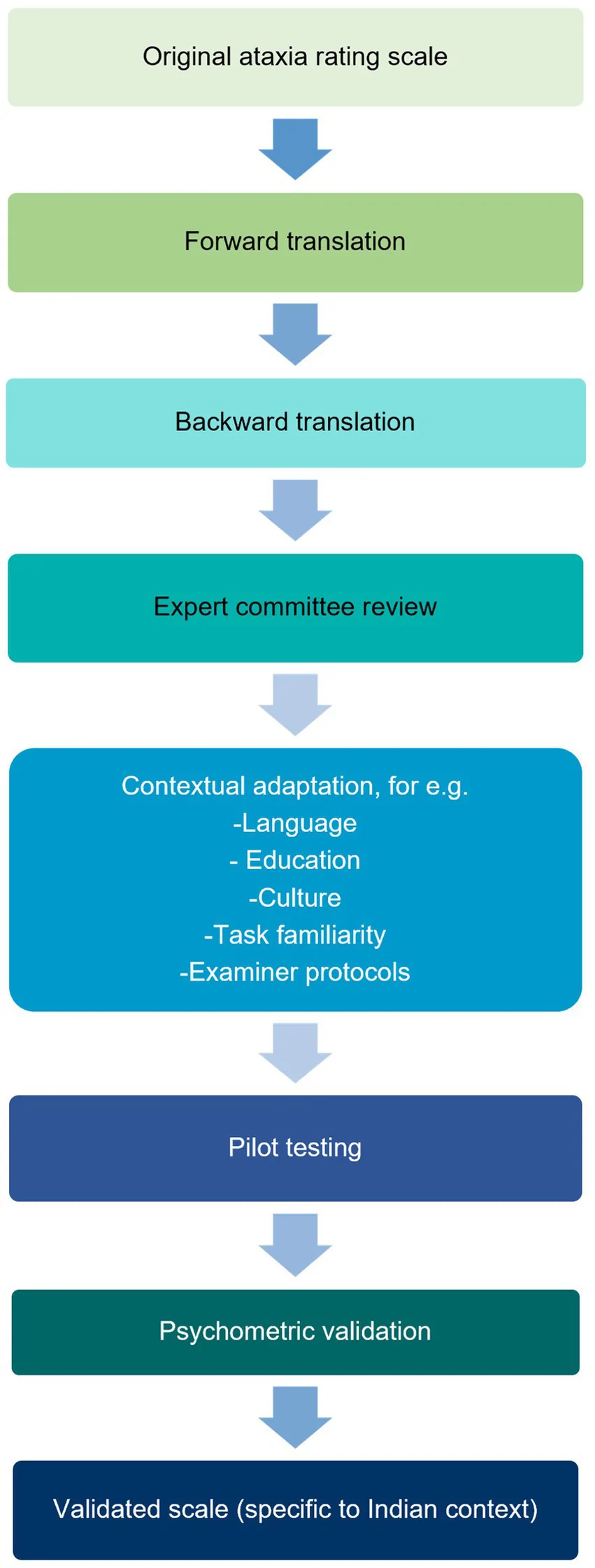
Framework for contextual validation of clinician-administered ataxia rating scales.

Digital technologies are increasingly being integrated into clinical practice and it would be crucial to evaluate whether the usage of wearable sensors and digital biomarkers in home and work environments can complement clinical scales while serving to reduce context-specific effects on outcome measurement. Another important aspect is that not all components of the scale may be equally susceptible to contextual influences. Tasks that depend on verbal instructions or examiner interpretation may be more variably interpreted as compared to motor tasks such as finger-nose testing. Future validation studies should evaluate both the total-scale and individual-item level measurement equivalence.

## Conclusion

Collectively, we illustrate that the administration and interpretation of clinician-rated ataxia scales may be influenced by factors beyond cerebellar dysfunction. Language may affect comprehension of assessment instructions, educational attainment may influence familiarity with task demands and cultural practices may alter the execution of certain performance-based assessments. Recognition of these contextual influences is relevant not only to the future adaptation and validation of SARA and ICARS, but also to the interpretation of existing multi-center datasets, harmonization of ongoing studies and development of future clinical outcome assessment strategies.

Neurological outcome measures should quantify cerebellar dysfunction rather than linguistic, educational, cultural, experiential differences or differences in attire. Cross-cultural adaptation should therefore extend beyond linguistic translation to encompass conceptual and task equivalence together with psychometric validation. A robust adaptation process should include forward and backward translation, expert committee review, cognitive debriefing, pilot testing and comprehensive evaluation of measurement properties (4, 20, 21). The adapted instrument should demonstrate adequate reliability (inter- and intra-rater reliability), construct and criterion validity, responsiveness to clinical change and measurement invariance to confirm equivalence with the original scale. Validation should follow established methodological standards for the translation, cultural adaptation and psychometric evaluation of clinician- and performance-based outcome measures, supplemented where appropriate by established measurement frameworks such as COSMIN (22, 23).

Without such evaluation, differences in scores may reflect contextual factors rather than true disease severity, thereby limiting longitudinal monitoring, multi-center studies and international clinical trials. Although this viewpoint focuses on India, the principles discussed are equally applicable to other multilingual and culturally diverse settings where clinician-administered neurological outcome measures are increasingly employed. Nevertheless, India’s exceptional linguistic, educational and cultural diversity provides a particularly compelling case for the systematic adaptation and psychometric validation of SARA and ICARS. While this viewpoint focuses on SARA and ICARS, the principles outlined are applicable across the spectrum of clinician-administered outcome measures for other neurological disorders as well. The objective of cross-cultural adaptation should therefore not be to make a scale linguistically familiar, but to ensure that the same neurological impairment produces an equivalent measurement regardless of the linguistic, educational, cultural or experiential context in which it is assessed. Such efforts will be essential to support equitable patient care, high-quality multi-center research and robust evaluation of emerging therapeutic interventions across diverse populations.

## Data Availability

The original contributions presented in the study are included in the article/supplementary material, further inquiries can be directed to the corresponding author.
